# Multisubject Learning for Common Spatial Patterns in Motor-Imagery BCI

**DOI:** 10.1155/2011/217987

**Published:** 2011-10-11

**Authors:** Dieter Devlaminck, Bart Wyns, Moritz Grosse-Wentrup, Georges Otte, Patrick Santens

**Affiliations:** ^1^Electrical Energy, Systems and Automation, Ghent University, Technologiepark 913, Zwijnaarde, 9052 Gent, Belgium; ^2^Department of Empirical Inference, Max Planck Institute for Biological Cybernetics, Spemannstr. 38, 72076 Tübingen, Germany; ^3^P.C. Dr. Guislain, Fr. Ferrerlaan 88A, 9000 Gent, Belgium; ^4^Department of Neurology, Ghent University Hospital, De Pintelaan 185, 9000 Gent, Belgium

## Abstract

Motor-imagery-based brain-computer interfaces (BCIs) commonly use the common spatial pattern filter (CSP) as preprocessing step before feature extraction and classification. The CSP method is a supervised algorithm and therefore needs subject-specific training data for calibration, which is very time consuming to collect. In order to reduce the amount of calibration data that is needed for a new subject, one can apply multitask (from now on called multisubject) machine learning techniques to the preprocessing phase. Here, the goal of multisubject learning is to learn a spatial filter for a new subject based on its own data and that of other subjects. This paper outlines the details of the multitask CSP algorithm and shows results on two data sets. In certain subjects a clear improvement can be seen, especially when the number of training trials is relatively low.

## 1. Introduction

 The development of BCI systems is an active research domain that has the goal to help people, suffering from severe disabilities, to restore the communication with their environment through an alternative interface. Such BCI systems can be divided in several categories based on the signal features they use. Some of these features like the P300 [1] and steady-state visual evoked potentials (SSVEPs) [2] are elicited naturally by external stimuli while others like the sensorimotor rhythms (SMRs) can be independently modulated by the subject. In case of SMR, this can be achieved by performing the task of imagining different movements, such as left and right hand movement, or foot and tongue movement. The cortical areas involved in motor function (and also motor imagery) show a strong 8–12 Hz (or even 18–26 Hz) activity when the person is not performing any motor (imagery) task. However, when the person is engaged in a motor task, the neural networks in the corresponding cortical areas are activated. This blocks the idle synchronized firing of the neurons and thus causes a measurable attenuation in those frequency bands. This decrease in power is also called event-related desynchronization (ERD) [3], the opposite effect is termed event-related synchronization (ERS). The location (electrode) of this feature depends on the type of motor task. For example, if a person moves his left arm, the brain region contralateral to the movement (around electrode C4) will display this ERD feature, while the intracellular potentials of the neurons in the ipsilateral cortical motor area continue to oscillate more synchronously.

Because of the low spatial resolution of electroencephalography (EEG), a commonly used method to improve this resolution is the common spatial pattern (CSP) algorithm introduced by Koles [4] to detect abnormal EEG activity. Later, it was used for discrimination of imagined hand movement tasks [5, 6]. Since then, a lot of groups improved the basic CSP algorithm by extending it with temporal filtering [7], making it more robust against nonstationarities [8] or reducing calibration time by transferring knowledge learned during previous sessions [9]. After more than a decade, this method still proves its superiority judging from the results of the fourth BCI competition (on http://www.bbci.de/competition/iv/ you can find the data sets and results of the 4th BCI competition). Still, this BCI setup is less accurate than the P300-based BCI and initially needs a longer training time. Some people are even unable to achieve proper control.

One way to further improve a subject-specific CSP filter is to use the data recorded from other subjects, additionally to the subject's own data. To this end, we will use some ideas of multisubject learning, an active topic in machine learning [10, 11]. In [12], the authors employed this concept to learn a classifier that was able to learn from multiple subjects, leading to an algorithm that performed well on new subjects even without training. The classifier could then be adapted when new data became available, reaching even higher classification accuracies with very few training samples. However, they applied a Laplacian filter instead of a spatial filter based on the CSP algorithm and used features obtained from the EEG signal after filtering it in distinct pass-bands. In contrast to their approach, we will start from the basic CSP algorithm and apply the multisubject learning concept to the preprocessing phase. In general, multisubject learning algorithms assume that all tasks are similar to each other. In our first approach, we will also assume that all subjects have similar head models and thus that the spatial filters can be decomposed into a subject-specific part and one global part. In a second approach, we will not make that assumption, but instead we will assume that they are grouped together in a fixed number of clusters. Furthermore, we include parameters to make a trade-off between these global and subject-specific filters.

Section 2 gives the details of the first approach of our multisubject CSP algorithm, while Section 4 presents the cluster-based multisubject CSP algorithm.  Section 3 presents an optimization framework for clustering CSP filters, which will also be used in the subsequent Section 4. The results are then compared with the basic CSP algorithm in Section 5 on one simulated data set and two experimental data sets, one of which is publicly available on the website of the third BCI competition [13] and one which includes data of 14 subjects recorded at the Max Planck Institute for Biological Cybernetics.  Section 6 highlights the strengths and the weaknesses of the method.

## 2. Multisubject CSP Formulation as a Sum of Convex-to-Convex Ratios

 The goal of the basic CSP method is to learn a set of spatial filters for one subject that maximizes the signal variance for trials of one class while at the same time minimizes the signal variance for trials of the other classes. For the two-class case, this can be formulated as follows:

(1) $$\underset{w}{\max}\;\frac{w^{T}\Sigma^{\left(1\right)}w}{w^{T}\Sigma^{\left(2\right)}w},$$

where Σ^(1)^ and Σ^(2)^ correspond to the covariance matrices of the trials corresponding to the first and second class, respectively.

We now want to use data of other subjects to improve the filters for specific subjects. To accomplish this, we first need a spatial filter **w**_*s*_ for each subject, which we decompose into the sum of a global and subject-specific part,

(2) $$w_{s}=w_{0}+v_{s},$$

where **w**_0_ ∈ ℝ^*d*^ represents the global spatial filter which is shared and learned over all subjects and **v**_*s*_ ∈ ℝ^*d*^ represents the subject-specific part of the filter. The number of channels is represented by *d*. A single optimization framework is proposed in which we learn both types of filters. This can be formulated as

(3) $$\underset{w_{0},v_{s}}{\max\;}\;\overset{S}{\underset{s=1}{\sum }}\frac{w_{s}^{T}\Sigma_{s}^{\left(1\right)}w_{s}}{w_{s}^{T}\Sigma_{s}^{\left(2\right)}w_{s}+\lambda_{1}{||w_{0}||}^{2}+\lambda_{2}{||v_{s}||}^{2}},$$

where the number of subjects is denoted by *S*.

The parameters *λ*_1_ and *λ*_2_ enable us to make a trade-off between the global or specific part of the filter. For a high value of *λ*_1_ and a low value of *λ*_2_, the vector **w**_0_ is forced to zero and a specific filter is constructed. When *λ*_2_ is high and *λ*_1_ low, the vector **v**_*s*_ is forced to zero and more global filters are computed. Furthermore, one can also perform regularization by choosing both *λ*_1_ and *λ*_2_ high.

The above equation can be rewritten to a simpler form, that is, a sum of convex-to-convex ratios

(4) $$\underset{w}{\max}\;R\left(w,\lambda \right)=\underset{w}{\max}\;\overset{S}{\underset{s=1}{\sum }}r_{s}=\underset{w}{\max}\;\overset{S}{\underset{s=1}{\sum }}\frac{w^{T}{\overset{̅}{\Sigma }}_{s}^{\left(1\right)}w}{w^{T}{\overset{̅}{\Sigma }}_{s}^{\left(2\right)}w},$$

with

(5) $$\begin{array}{c} w^{T}=\left(\begin{array}{cccc} w_{0}^{T} & v_{1}^{T} & \cdots & v_{S}^{T} \end{array}\right),\\ {\overset{̅}{\Sigma }}_{s}^{\left(1\right)}=E_{s}\Sigma_{s}^{\left(1\right)}E_{s}^{T},\\ {\overset{̅}{\Sigma }}_{s}^{\left(2\right)}=E_{s}\Sigma_{s}^{\left(2\right)}E_{s}^{T}+\lambda_{1}D_{0}+\lambda_{2}D_{s},\\ E_{s}=\left(\begin{array}{c} I_{d\times d}\\ 0_{(s-1)d\times d}\\ I_{d\times d}\\ 0_{(S-s)d\times d} \end{array}\right),\\ D_{0}=\left(\begin{array}{c} I_{d\times d}\\ 0_{Sd\times d} \end{array}\right)\left(\begin{array}{cc} I_{d\times d} & 0_{d\times Sd} \end{array}\right),\\ D_{s}=\left(\begin{array}{c} 0_{sd\times d}\\ I_{d\times d}\\ 0_{(S-s)d\times d} \end{array}\right)\left(0_{d\times sd}\;I_{d\times d}\;0_{d\times (S-s)d}\right), \end{array}$$

where *I*_*d*×*d*_ represents the *d*-dimensional unity matrix.

To find the maximum of (4) we use a Newton method. To this end, we need both the gradient and Hessian of (4). The gradient is given by,

(6) $$\nabla_{w}R\left(w,\lambda \right)=2\overset{S}{\underset{s=1}{\sum }}\frac{{\overset{̅}{\Sigma }}_{s}^{\left(1\right)}w-r_{s}\left(w\right){\overset{̅}{\Sigma }}_{s}^{\left(2\right)}w}{w^{T}{\overset{̅}{\Sigma }}_{s}^{\left(2\right)}w},$$

while the Hessian is given by,

(7) $$\begin{array}{cc} \nabla_{w}\left(\nabla_{w}R\right) & =2\overset{S}{\underset{s=1}{\sum }}\\ & \;\;[\frac{\left({\overset{̅}{\Sigma }}_{s}^{\left(1\right)}-r_{s}{\overset{̅}{\Sigma }}_{s}^{\left(2\right)}-\nabla_{w}^{\left(s\right)}w^{T}{\overset{̅}{\Sigma }}_{s}^{\left(2\right)}\right)}{\delta_{s}}\\ & \;\;\;-\frac{\left({\overset{̅}{\Sigma }}_{s}^{\left(1\right)}w-r_{s}{\overset{̅}{\Sigma }}_{s}^{\left(2\right)}w\right)w^{T}{\overset{̅}{\Sigma }}_{s}^{\left(2\right)}}{\delta_{s}^{2}}], \end{array}$$

where *δ*_*s*_ is short for the denominator of the term *r*_*s*_ and ∇_**w**_^(*s*)^ for the gradient of *r*_*s*_ with respect to **w**.

From here on, this method is denoted by the abbreviation “mtCSP.”

## 3. An Optimization Framework for Clustering Spatial Filters

Before giving the details of the cluster-based multisubject CSP algorithm, we present an optimization algorithm for clustering CSP filters. This algorithm is inspired by [14] and will form the basis of the algorithm described in the next section. It will also be employed to find a good initialization for the variables in the cluster-based multisubject CSP algorithm.

So, let us start with a simplified version of the optimization framework proposed in [14]

(8) $$\underset{\alpha_{sk},\mu_{k}}{\min}\overset{K}{\underset{k=1}{\sum }}\overset{S}{\underset{s=1}{\sum }}\alpha_{sk}d\left(\mu_{k},x_{s}\right),$$

(9) $$\alpha_{sk}\in \left\{0,1\right\},\;\;\overset{K}{\underset{k=1}{\sum }}\alpha_{sk}=1,$$

where *K* is the number of clusters, *S* the number of observations, **x**_*s*_ the observations, ***μ***_*k*_ the cluster centers and *d* a distance function. The binary coefficient *α*_*sk*_ indicates the cluster to which a certain object belongs. This minimization is typically solved by cycling through two steps. In a first step, the coefficients *α*_*sk*_ are determined by setting the *k*th coefficient to one if the object **x**_*s*_ lies closest to the cluster center ***μ***_*k*_

(10) $$\alpha_{sk}=\{\begin{array}{cc} 1, & \text{if}\;d\left(\mu_{k},x_{s}\right)<d\left(\mu_{l},x_{s}\right),\\ & \;\;\;\forall l\in \left\{1,\ldots ,K\right\}\setminus k,\\ 0, & \text{otherwise}. \end{array}$$

In the second step, we find the cluster centers that minimize the total distance to their cluster members as determined by the coefficients *α*_*sk*_ computed in the previous step. Given the coefficients *α*_*sk*_, we can see that the inner sums are independent of each other and thus can also be optimized independently of each other. A typical distance function is the Euclidean distance.

For spatial filters, however, we have to find a more appropriate metric. As explained in [9], the space of CSP filters is not Euclidean. Changing the length or the sign of a CSP filter does not matter as it is still a solution of the Rayleigh quotient (1). In other words, the filters can all be considered to lie on the unit hypersphere and thus we employ an angle-based metric instead. This metric should be zero when the angle between two spatial filters is zero or *π* radials and maximal when *π*/2 radials. Consequently, the squared sine of the angle *θ* between the two filters seems an appropriate metric

(11) $$\begin{array}{cc} d\left(v_{1},v_{2}\right) & =\sin^{2}\left(\theta \right)=1-\cos^{2}\left(\theta \right)\\ & =1-\frac{{\left(v_{1}^{T}v_{2}\right)}^{2}}{\left(v_{1}^{T}v_{1}\right)\left(v_{2}^{T}v_{2}\right)}. \end{array}$$

We can now plug this expression in (8) and drop the constant one as it does not change the solution of the optimization problem. The sign can also be dropped if we transform (8) into a maximization problem, resulting in,

(12) $$\begin{array}{c} \underset{\alpha_{sk},w_{k}}{\max}\overset{K}{\underset{k=1}{\sum }}\overset{S}{\underset{s=1}{\sum }}\alpha_{sk}\frac{{\left(w_{k}^{T}v_{s}\right)}^{2}}{\left(w_{k}^{T}w_{k}\right)\left(v_{s}^{T}v_{s}\right)},\\ \alpha_{sk}\in \left\{0,1\right\},\;\;\overset{K}{\underset{k=1}{\sum }}\alpha_{sk}=1, \end{array}$$

where **w**_*k*_ represents the *k*th cluster center. In the second step of the algorithm, we have to find the optimal cluster centers **w**_*k*_ and this can be done independently for each cluster (and thus each inner sum). Under the assumption that **v**_*s*_^*T*^ **v**_*s*_ = 1, this inner sum for cluster *k* can then be rewritten as

(13) $$\frac{w_{k}^{T}\left(\sum_{s\in S_{k}}^{}v_{s}v_{s}^{T}\right)w_{k}}{w_{k}^{T}w_{k}},$$

where *S*_*k*_ is the set of all filters that belong to the *k*th cluster. This expression has to be maximized with respect to **w**_*k*_. The maximum is simply the principal component of the covariance matrix of filters within the cluster *k* and thus equals the eigenvector with the largest eigenvalue of the corresponding eigenvalue decomposition.

## 4. Cluster-Based Multisubject CSP

In Section 2, we assumed that all subjects were similar. This assumption should off course be relaxed. Here, we present an algorithm that groups similar subjects together in clusters. Cross-subject learning is then performed on each of the separate clusters. The method is inspired by the optimization algorithm as described in Section 3.

First, we introduce multiple shared filters **w**_*k*_, one for each cluster *k*,

(14) $$w_{sk}=w_{k}+v_{sk}.$$

We can now transform problem (8) to a maximization problem and replace the distance function with a quotient similar to the one in (3), resulting in the following formulation:

(15) $$\begin{array}{c} \underset{\alpha_{sk},w_{k},v_{sk}}{\max}\overset{K}{\underset{k=1}{\sum }}\overset{S}{\underset{s=1}{\sum }}\alpha_{sk}\frac{w_{sk}^{T}\Sigma_{s}^{\left(1\right)}w_{sk}}{w_{sk}^{T}\Sigma_{s}^{\left(2\right)}w_{sk}+\lambda_{1}{||w_{k}||}^{2}+\lambda_{2}{||v_{sk}||}^{2}},\\ \alpha_{sk}\in \left\{0,1\right\},\;\;\overset{K}{\underset{k=1}{\sum }}\alpha_{sk}=1. \end{array}$$

In the first step, the coefficients *α*_*sk*_ can again be determined in a similar manner

(16) $$\alpha_{sk}=\{\begin{array}{cc} 1, & \text{if}\;\frac{w_{sk}^{T}\Sigma_{s}^{\left(1\right)}w_{sk}}{w_{sk}^{T}\Sigma_{s}^{\left(2\right)}w_{sk}+\lambda_{1}{||w_{k}||}^{2}+\lambda_{2}{||v_{sk}||}^{2}}\\ & >\frac{w_{sl}^{T}\Sigma_{s}^{\left(1\right)}w_{sl}}{w_{sl}^{T}\Sigma_{s}^{\left(2\right)}w_{sl}+\lambda_{1}{||w_{l}||}^{2}+\lambda_{2}{||v_{sl}||}^{2}},\\ & \;\;\;\;\;\;\forall l\in \left\{1,\ldots ,K\right\}\setminus k,\\ 0, & \text{otherwise}. \end{array}$$

In the second step, we apply the multisubject CSP algorithm as discussed in Section 2, maximizing the inner sum of (15) with respect to **w**_*k*_ and **v**_*sk*_ for subjects belonging to the respective cluster *k*. This completes the two steps of the algorithm. There is, however, still a small problem with the first step as the subject-specific vectors **v**_*sl*_ are unknown for subjects belonging to cluster *k*. This is because in the second step we compute **v**_*sk*_ only for subjects belonging to the *k*th cluster. To this end, we still have to optimize the quotient in (15) for each subject separately with respect to **v**_*sl*_ and fixed **w**_*l*_ for each *l* ≠ *k* (*k* representing the cluster to which the subject belongs).

Finally, we also want to find a good initialization for the variables. To accomplish this, we use the clustering algorithm described in Section 3 and apply it on the subject-specific filters as computed with the basic CSP algorithm. This gives us an initial estimation of the cluster coefficients *α*_*sk*_. We can also use the cluster centers and the difference between them and the subject-specific filters to initialize **w**_*k*_ and **v**_*sk*_, respectively.

## 5. Experiments

### 5.1. Simulated Data

For the simulated data we generate two clusters of 20 similar tasks. The training set of each task contains data for two conditions, each condition counting 15 samples. The source variables are generated from a two-dimensional Gaussian distribution with zero mean and covariance matrix dependent on the condition, but the same for both clusters and all tasks,

(17) $$\Sigma^{\left(1\right)}=\left(\begin{array}{cc} 5 & 0\\ 0 & 1 \end{array}\right),\;\;\Sigma^{\left(2\right)}=\left(\begin{array}{cc} 1 & 0\\ 0 & 5 \end{array}\right).$$

The columns of the mixing matrices are also generated from a two-dimensional Gaussian, parameterized by an isotropic covariance matrix of low variance (1 × 10^−4^). The means are fixed and different for the two clusters, but the same for all tasks in the same cluster

(18) $$\begin{array}{cc} A_{1} & =\left(\begin{array}{cc} 0.3500 & 0.6062\\ -1.0392 & 0.6000 \end{array}\right),\\ A_{2} & =\left(\begin{array}{cc} 0.6657 & 0.2163\\ -0.3708 & 1.1413 \end{array}\right). \end{array}$$

**Figure 1:**
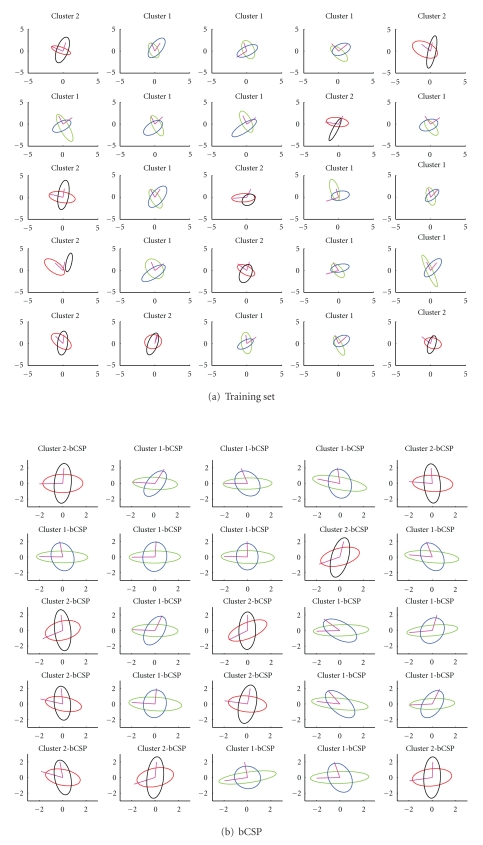

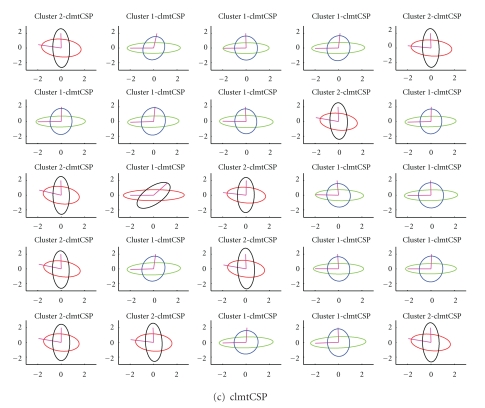
(a) shows the training set that is used to compute both the bCSP and clmtCSP filters. The data points themselves are not plotted, instead we only draw the standard deviation contours of the data's estimated covariance matrix, together with its corresponding principal vectors (representing the ellipse's principal axis). Blue and black contours correspond to the first class or condition, while green and red contours represent the other class. The goal of the computed filters is to align the principal vectors to the axes. The results for both bCSP and clmtCSP are shown in (b) and (c) figures, respectively. Here, the contours denote the standard deviations according to the estimated covariance matrix of the “unmixed” sources. Concerning the clmtCSP method, if the contours are drawn in blue and green, it means that they have been estimated as being in the first cluster according to the algorithm. If it is red and black, the task is estimated as belonging to the second cluster. The true cluster number is given in the title of each subplot.

**Figure 2:**
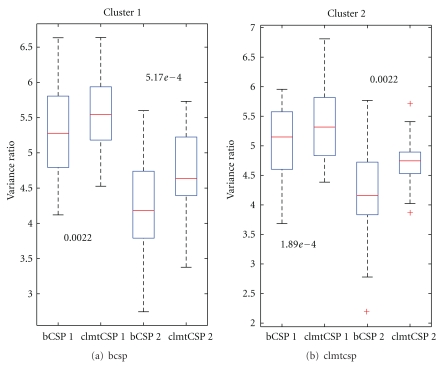
(a) compares the variance ratios of the bCSP solution with the clmtCSP solution on the first cluster, while (b) makes the comparison for tasks of the second cluster. The number above or below each pair of bars is the *P*  value according to the paired Wilcoxon signed rank test. The numeric suffix on the tick labels of the *x*-axis denotes the source number.

We also add some noise with zero mean and very low variance (1 × 10^−3^) to the mixed observations. A sample training set is displayed in Figure 1(a). A similar test set is created with 285 data points for each of the conditions. We then apply the basic CSP (bCSP) method on each of these tasks separately and compare it with the clustered multisubject version (clmtCSP). The basic CSP solution is shown for the first 25 (out of 40) tasks in Figure 1(b). The final solution of the clustered multisubject learning method is shown in Figure 1(c). In this toy example, we do not perform a preclustering on the specific filters to find a good initialization. Instead, the first 20 tasks are considered (or initialized) to belong to the first cluster and the last 20 tasks are considered to belong to the second cluster. This way, we can check how well the algorithm is able to find the correct clusters. Figure 1(c) tells us that the algorithm is quite able to assign the tasks to the correct clusters. It is, however, not perfect by any means as you can see for the task in the third row and second column. Furthermore, one can see that the principal axis of the ellipses are better aligned after application of the clmtCSP algorithm compared to the bCSP solution. To quantify the difference between the two methods, we compute the variance ratios of the estimated sources (unmixed observations) which results in

(19) $$\frac{\max\left({\hat{\Sigma }}_{11}^{\left(1\right)},{\hat{\Sigma }}_{11}^{\left(2\right)}\right)}{\min\left({\hat{\Sigma }}_{11}^{\left(1\right)},{\hat{\Sigma }}_{11}^{\left(2\right)}\right)},\;\;\frac{\max\left({\hat{\Sigma }}_{22}^{\left(1\right)},{\hat{\Sigma }}_{22}^{\left(2\right)}\right)}{\min\left({\hat{\Sigma }}_{22}^{\left(1\right)},{\hat{\Sigma }}_{22}^{\left(2\right)}\right)},$$

for each source, respectively. These ratios are calculated for both clusters. Because the sources can be switched and the order is not necessarily the same for both methods, we sort the ratios from high to low. The two highest ratios of both methods are then compared with each other, as are the two lowest. These results are summarized per cluster in the boxplot of Figure 2. We can see that the medians of the ratios are always larger for the clmtCSP method. A paired Wilcoxon signed rank test rejects the hypothesis of equal medians for both sources and both clusters. The corresponding *P*  values are also given in Figure 2. 

### 5.2. Experimental Data Sets

 For the experimental data sets we use data of the third BCI competition (BCIC3 data set (on http://www.bbci.de/competition/iii/ you can find the data sets and results of the 3e BCI competition), more precisely data set IVa and a data set of 14 subjects recorded at the Max Planck Institute (MPI data set) for Biological Cybernetics.

The set of the BCI competition contains data recorded from 118 electrodes where the subjects performed two tasks: right hand motor imagery and foot imagery. Five subjects are included in the set and each subject recorded 280 trials. We take a fixed test set of the last 180 trials while the first 100 are retained to construct the training sets. To limit the number of parameters that needs to be computed by the optimization algorithm, the number of channels is reduced to 22. The ones selected are Fp1, Fpz, Fp2, F7, F3, Fz, F4, F8, T7, C3, Cz, C4, T8, P7, P3, Pz, P4, P8, POz, O1, Oz and O2.

In the MPI set, each subject performed 30 left hand motor imagery trials and 30 right hand motor imagery trials. This was repeated once for the test set resulting in a total of 120 trials per subject. The same subset of electrodes is used as before except for two channels which were not recorded for some of the subjects.

As there are only five subjects in the BCIC3 data set, we assume that all subjects are similar. Consequently, we will simply apply the first proposed algorithm, that is, mtCSP. The MPI data set, however, contains too many subjects to assume that they are all similar. Hence, we will apply the cluster-based “clmtCSP” method with a predefined number of clusters, namely, three. Four cluster seems too many for only 14 subjects, as this could potentially leave some clusters with very few subjects. On the other hand, we did not choose two for reasons of complexity as it increases the number of subjects per cluster and thus the dimensionality of problem (4). At this stage, the optimization algorithms to solve the nonconvex problem (4) are not sufficient for such high dimensions.

All signals are band-pass filtered between 8 and 30 Hz. The trade-off parameters *λ*_1_ and *λ*_2_ are determined through 5-fold cross validation. For each subject, only two spatial filters are computed: one for each class. Cross-validation is done for the following set of parameters: *λ*_1_, *λ*_2_ ∈ {10^−4^, 10^−3^, 10^−2^, 10^−1^, 1,10^1^, 10^2^, 10^3^, 10^4^}. The performance on each fold is measured by the average accuracy (over all subjects) of the linear discriminant (LDA) classifier on that fold. Given the known good performance of LDA in motor-imagery experiments, we not only use it for scoring each fold, but also as the final classifier.

**Figure 3:**
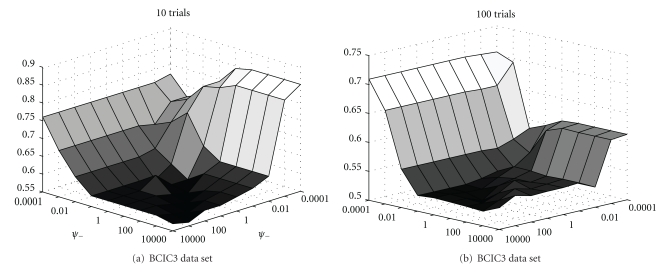
Cross-validation accuracies per parameter combination of *λ*_1_ and *λ*_2_ on the BCIC3 data set. We performed 5-fold cross-validation per subject. Averaging the result over all folds and all subjects gives the final result as plotted in the figure.

Figure 3 gives some cross-validation plots on the BCIC3 set for the mtCSP algorithm, showing the average accuracy (over subjects and folds) for each parameter setting. It is clear that for a lower number of training trials (10 per class), the parameters values are biased towards the promotion of shared filter components, while penalizing the subject-specific components. For more training trials (about 100 per class), it is clear that the parameters values tend to be subject specific. For the MPI data set, we fix *λ*_1_ = 0 to lower the computational demands. According to Figure 3, this seems to be a good choice as the parameter values at the boundary of the grid produce the most interesting results. Furthermore, the line defined by fixing *λ*_1_ = 0.0001 displays most variability, while the line defined by fixing *λ*_2_ = 0.0001 does not seem to indicate much change when *λ*_1_ is varied.

The results for each subject separately are given in Tables 1 and 2 for both the BCIC3 and MPI data set, respectively. The header of each table presents the values of the parameters *λ*_1_ and/or *λ*_2_ as determined through cross-validation. Also note that the mean is computed only on those subjects for which one of the methods at least achieves above chance level (with 180 trials in the test set, we can fix the chance level at an accuracy of 56% for the BCIC3 set. For the MPI data set, we set the chance level at 60%) accuracies.

The first thing we notice for the BCIC3 set is that for 5 trials (from here on, we state the number of training trials per class, e.g., when we mention 5 training trials, we mean 5 trials per class, thus 10 in total.) The impact of the multisubject version is relatively low, although this is the area where we suspected the impact would be the largest. Nevertheless, for some subjects, like subject *aa* the impact is substantial as it goes from chance level to an accuracy well above 70%. On the other hand, there is subject *aw* where the accuracy drops to chance level when employing the multisubject version. This subject, however, never seems to benefit from the multisubject learning. These two subjects can give us some insight in to the reason of the failure, which we attribute to the way we determine the parameters *λ*_1_ and *λ*_2_. This is done globally across all subjects and consequently the values are taken the same for all. Obviously, these parameters should be determined for each new subject separately. The ideal case would thus be to include five trials of the “new” subject's training set, all training trials of the other subjects and repeat this process for each subject. On the other hand, this would require us to determine the parameters per subject independently on a set of only five trials per class, which is prone to be unstable.

The difference between both methods becomes apparent in the case of ten training trials where the mtCSP method achieves better or equal accuracies compared to the bCSP method on all subjects, except again subject *aw*. As there are only five subjects, we are not able to show the difference is significant with a paired Wilcoxon signed rank test.

Table 2 shows the results for the cluster-based mtCSP method on all 14 subjects. Looking at chosen parameter values for *λ*_2_, we can see that subject-specific filter components are most penalized when only five training trials are available, while they are least penalized when 20 training trials are available. This is reflected in the results as there is almost no difference between bCSP and clmtCSP in case of 20 training trials. However, there is quite some difference between the two methods for five trials. Unfortunately, a paired Wilcoxon signed rank test (only considering those subjects for which one of the methods performs above chance level) does not indicate a significant difference. Note that (in case of five training trials) only eight subjects are included in the test.

## 6. Discussion and Future Work

 We presented a multisubject extension to the basic CSP algorithm in order to reduce the number of training trials and to improve performance by learning spatial filters across subjects. It involves a nonconvex optimization problem and thus a global solution is not guaranteed when employing standard optimization techniques. However, the optimization of such a sum of convex to convex ratios is a hot topic in optimization theory. We can expect, that in the future, implementations will come available that guarantee global convergence and are scalable to handle high-dimensional problems. The authors in [15] present such solution for seemingly small-sized problems.

The main downside of the proposed methods is that we have to perform cross-validation to select good parameter values. Firstly, this takes time to compute, rendering the methods impractical as one can record more data within that time frame to compute good filters. Secondly, enough data needs to be available to determine the parameter values through cross-validation. This is of course in contrast with the aim of the proposed algorithms to reduce the number of training trials. In order to find indicators for the potential of the methods on a low number of training trials, we performed cross-validation by averaging scores over several folds and subjects. This leads to more stable and reliable estimates of the parameter values. We then choose the parameter values the same for all subjects. However, the need for cross-validation could be avoided by employing the Bayesian framework. In order to learn a model across several subjects in this framework, the use of shared priors will be the topic of future research.

An open question is how it compares to other CSP variants that learn from other subjects [16]. The latter method computes the filters by combining the covariance matrices of several subjects instead.

Due to the way we perform cross-validation, it is impossible to show the method's true potential. Nevertheless, some of the results indicate that (cluster-based) multisubject learning for CSP leads to a noticable improvement for some subjects. That some subjects suffer from these methods could be avoided if the trade-off parameters could be chosen reliably for each new subject separately with little training data.

Finally, we want to add that this manner of including the clustering in the optimization problem may be employed for cluster-based multisubject classifiers too. Note that Fisher's discriminant analysis [17] can be written as a generalized Rayleigh quotient and thus be solved with a generalized eigenvalue decomposition, similar to CSP. Instead of using the quotient of (3), we could plug in a modified version of Fisher's ratio.

## Figures and Tables

**Table 1:** Classification accuracies per subject for the BCI competition data set.

	5 trials		10 trials		20 trials		30 trials	
	*λ*_1_ = 10^−3^, *λ*_2_ = 10^−1^		*λ*_1_ = 10^−4^, *λ*_2_ = 10^1^		*λ*_1_ = 10^−3^, *λ*_2_ = 10^−1^		*λ*_1_ = 10^−4^, *λ*_2_ = 10^1^	
Subject	bCSP	mtCSP	bCSP	mtCSP	bCSP	mtCSP	bCSP	mtCSP
*aa*	0.49	0.73	0.54	0.64	0.66	0.71	0.61	0.69
*al*	0.80	0.73	0.95	0.93	0.95	0.94	0.94	0.94
*av*	0.56	0.58	0.59	0.63	0.44	0.62	0.56	0.64
*aw*	0.69	0.57	0.69	0.56	0.66	0.58	0.55	0.54
*ay*	0.92	0.86	0.84	0.93	0.85	0.85	0.88	0.87

Mean	0.69	0.69	0.72	0.74	0.71	0.74	0.75	0.79

**Table 2:** Classification accuracies per subject for the MPI set.

	5 trials		10 trials		20 trials	
	*λ*_2_ = 10^−1^		*λ*_2_ = 10^−2^		*λ*_2_ = 10^−4^	
Subject	bCSP	clmtCSP	bCSP	clmtCSP	bCSP	clmtCSP
1	0.80	0.68	0.78	0.73	0.85	0.85
2	0.85	0.83	0.83	0.77	0.87	0.85
3	0.45	0.43	0.53	0.57	0.58	0.60
4	0.58	0.53	0.72	0.75	0.77	0.77
5	0.53	0.47	0.52	0.48	0.62	0.60
6	0.58	0.67	0.60	0.60	0.70	0.70
7	0.83	0.92	0.90	0.92	0.95	0.95
8	0.38	0.52	0.48	0.48	0.53	0.53
9	0.57	0.70	0.58	0.62	0.63	0.63
10	0.68	0.53	0.60	0.62	0.63	0.60
11	0.50	0.53	0.42	0.52	0.40	0.43
12	0.52	0.68	0.65	0.70	0.63	0.63
13	0.62	0.60	0.63	0.58	0.57	0.60
14	0.53	0.53	0.50	0.47	0.55	0.57

Mean	0.68	0.70	0.70	0.70	0.71	0.71
